# Effect of Topical Hyaluronic Acid on Wound Healing and Pain Following Electrocautery Frenectomy: A Randomized Controlled Trial

**DOI:** 10.7759/cureus.106244

**Published:** 2026-03-31

**Authors:** B Roshan Arbaaz, S Senthil Nathan, Sankaralingam Thirumalai, Karunanithi Arunagiri, Guna Harini, V Vijaykumar, Thangadurai Maheswaran

**Affiliations:** 1 Periodontics, Sri Venkateshwaraa Dental College, Pondicherry, IND; 2 Periodontics, Mahatma Gandhi Postgraduate Institute of Dental Sciences, Pondicherry, IND; 3 Orthodontics, Adhiparasakthi Dental College and Hospital, Melmaruvathur, IND; 4 Oral and Maxillofacial Pathology, Adhiparasakthi Dental College and Hospital, Melmaruvathur, IND

**Keywords:** electrocautery, frenectomy, hyaluronic acid, labial frenum, landry healing index, oral soft tissue healing, postoperative pain, randomized controlled trial, visual analog scale, wound healing

## Abstract

Introduction: Aberrant labial frenal attachments can lead to plaque accumulation, gingival inflammation, periodontal deterioration, and aesthetic issues. Electrocautery frenectomy provides effective hemostasis; however, it may result in delayed wound healing due to thermal tissue damage. Hyaluronic acid (HA) has demonstrated anti-inflammatory and pro-healing properties in the management of oral soft tissues. This study aimed to assess wound healing and postoperative pain following electrocautery frenectomy with and without the topical application of HA gel.

Methods: This randomized controlled clinical trial included 24 systemically healthy participants (aged 18-60 years) with type III or type IV maxillary labial frenal attachments. Participants were randomly assigned to two groups: Group 1 (test group) underwent electrocautery frenectomy followed by the topical application of 0.2% HA gel (Gengigel®, Ricerfarma S.r.l., Milano, Italy) for three days postoperatively, while Group 2 (control group) underwent electrocautery frenectomy alone. Wound healing was evaluated using the Landry et al. healing index at days 7, 14, 21, and 28 postoperatively. Postoperative pain was assessed using a visual analog scale (VAS) on days 1, 3, and 7. Intergroup comparisons were conducted using independent t-tests.

Results: The test group exhibited significantly superior wound healing scores compared to the control group at all postoperative time points (p<0.05). The mean VAS pain scores were significantly lower in the HA group on postoperative days 1, 3, and 7 (p<0.0001).

Conclusion: The topical application of HA gel following electrocautery frenectomy significantly enhances wound healing and reduces postoperative pain. HA can be considered a beneficial adjunct for improving postoperative outcomes after frenectomy procedures.

## Introduction

Pathological labial frenulum attachments cause diastema and muscle pull, resulting in plaque accumulation. Furthermore, the forceful tugging of muscle fibers over time may result in the formation of periodontal pockets, tooth loss, gingival recession, and papillary loss [1]. Poor control of plaque can lead to a more noticeable loss of periodontal tissue, particularly in the elderly. Regular brushing habits can also be challenging if there is a small sulcus as well as a high frenulum attachment. Many researchers have evaluated the effectiveness of electrocautery in frenectomy. With recent advancements in electrosurgical procedures, such as argon beam coagulation (ABC), complications, such as burns, explosions due to the use of flammable gases, interference with pacemakers, and emission of surgical smoke, have not yet been reported [2].

Electrocautery has been used since 1928. It is usually advised in non-compliant cases and in cases in which hemostasis is difficult to achieve. The main benefit of electrocautery is its coagulative effect, which provides a clear view of the surgical field. Any device that generates thermal energy to cut or ablate tissues releases some amount of heat via diffusion into nearby tissues (conduction) or convection into the bloodstream. Tissues that are thermally injured may not heal quickly and are more prone to dehiscence [3]. Various chemotherapeutic agents, such as chlorhexidine and povidone iodine, have been employed for wound healing. Various natural and commercially available wound care products have been investigated, including collagen, platelet-rich fibrin, cyanoacrylate, and hyaluronic acid (HA) [4].

HA has been used in recent years as an adjunct to improve the healing of oral soft tissues [5-8]. It belongs to the glycosaminoglycan family of nonsulfated polysaccharides with a high-molecular-weight polymer found in blood, gingival crevicular fluid, saliva, and synovial fluid, when compared with other bodily fluids. It plays a crucial role in the extracellular matrix of the epidermis, connective tissue, and synovial joints, among other tissues. HA exhibits pro-angiogenic, anti-inflammatory, fungistatic, and anti-edematous effects on tissues. These characteristics imply that HA may promote oral soft tissue wound healing, such as post-frenectomy procedures [9-11]. Hyaluronan synthase enzymes help in the synthesis of high-molecular-weight HA in the periodontium, which is later extensively broken down into lower-molecular-weight molecules. It is employed in surgical procedures because of its capacity to induce osteogenesis [12].

Gengigel® (Ricerfarma S.r.l., Milano, Italy), which comprises high-molecular-weight fractions of HA in a gel formulation with a 0.2% concentration, has been used in conjunction with scaling and root planing to treat plaque-induced gingivitis [13]. The safety and non-irritating properties of Gengigel® have been evaluated using skin irritation, sensitizing potentiality, and percutaneous absorption tests [14]. It has been proven that the hygroscopicity of 0.2% HA gel permits it to hold onto water and preserve conformational rigidity. In order to decrease inflammation and encourage wound healing, HA has been recommended as a monotherapy in periodontology or as an adjuvant to nonsurgical and/or surgical periodontal therapy [15]. Studies have shown that 0.8% hyaluronan, when used with mechanical debridement, significantly improves healing following nonsurgical therapy [16]. A study revealed that when Gengigel® was used during the first two months following implant placement (the healing/osteo-integration period), the outcomes significantly improved [17]. Topical application of HA has been proven to be efficacious in reducing postoperative pain and burning sensation and speeding up the healing time of the palatal wound with respect to epithelization and color match [18]. Thus, the current study was conducted to evaluate wound healing and postoperative pain with and without the use of topical application of HA gel following frenectomy performed using electrocautery.

## Materials and methods

Study design

This randomized controlled trial was conducted in the outpatient department of the Department of Periodontology at Sri Venkateshwaraa Dental College, Pondicherry, India, during September 2024 to January 2025. Ethical approval was obtained from the Institutional Ethical Committee of Sri Venkateshwaraa Medical College Hospital and Research Centre (approval number: 27/SVMCH/IEC-Cert/April 23), and the study was registered in the Clinical Trials Registry-India (CTRI) (registration number: CTRI/2024/08/073096). The ethical principles outlined in the Declaration of Helsinki were rigorously followed throughout the study.

Sample size calculation was performed using G*Power (Version 3.1; Heinrich-Heine-University, Düsseldorf, Germany). Considering an effect size of 1.4, an alpha error probability of 0.05, and a study power of 95%, the minimum required sample size was calculated to be 24 subjects (12 in each group) for detecting significant differences between two independent groups using an independent t-test.

Participants

**Figure 1:**
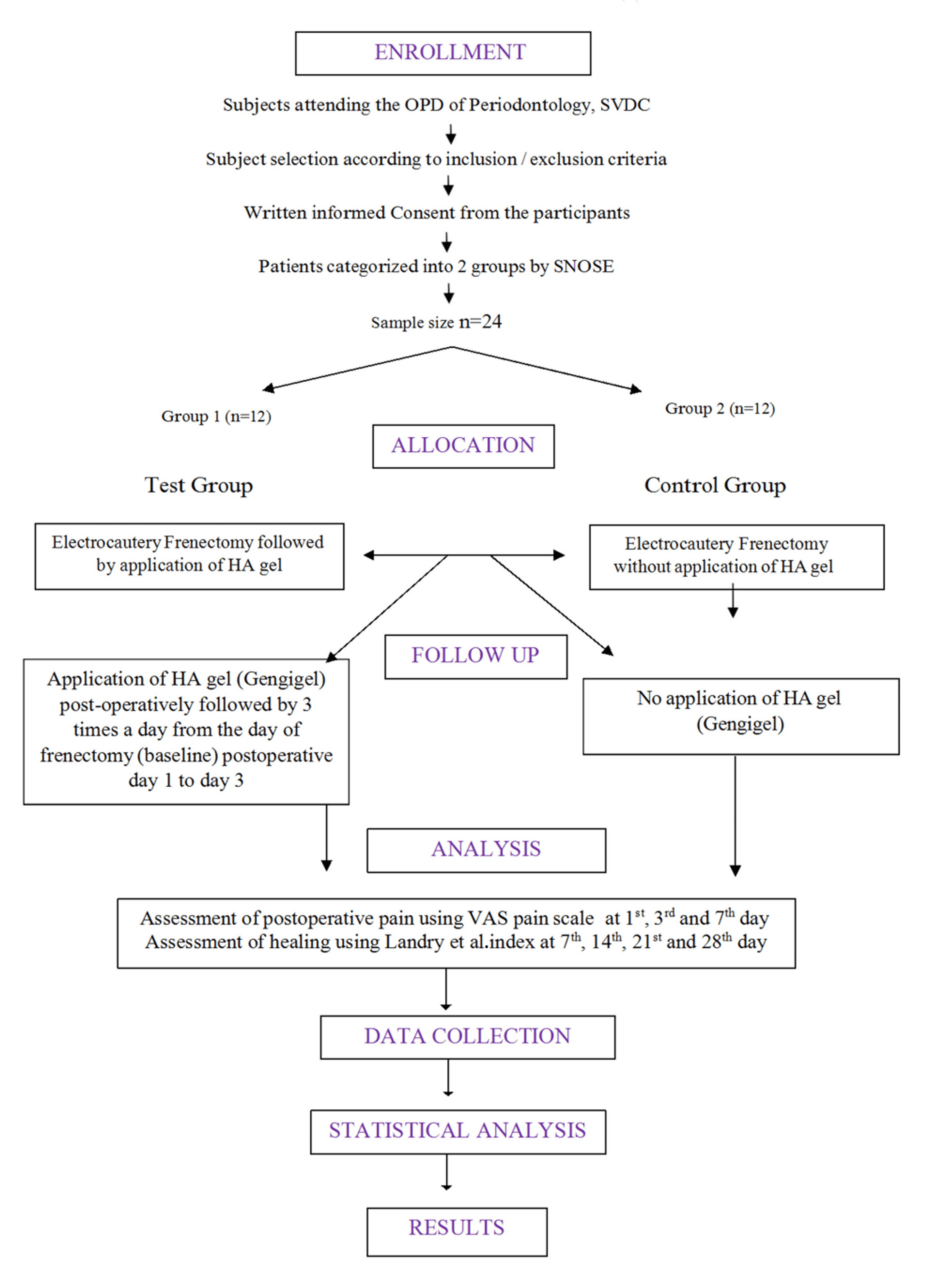
Methodology flowchart Flow diagram showing subject selection, allocation into test and control groups, follow-up, and final analysis of participants. OPD: Outpatient Department; SVDC: Sri Venkateshwaraa Dental College; SNOSE: Sequentially Numbered Opaque Sealed Envelope; HA: hyaluronic acid; VAS: visual analog scale

In total, 24 subjects who fulfilled the eligibility criteria were included in this study (Figure 1). The inclusion criteria were as follows: (1) subjects of both sexes within the age group of 18-60 years, (2) good oral hygiene at the time of surgery, (3) subjects with aberrant maxillary anterior frenum, (4) all subjects who were systemically healthy, and (5) type III and type IV frenal attachments, according to Mirko et al. [19]. The exclusion criteria were as follows: (1) poor oral hygiene, (2) patients with underlying systemic conditions, (3) chronic smokers, and (4) pregnant and lactating mothers. All subjects received a detailed information sheet about the study in their local language and provided written informed consent.

Randomization

The subjects were randomly divided into two groups using a computer-generated random sequence: Group 1 (test group) underwent electrocautery frenectomy followed by the application of HA gel (Gengigel®) from the day of frenectomy (baseline) postoperative days 1-3, while Group 2 (control group) underwent electrocautery frenectomy without the application of Gengigel® gel.

Allocation concealment was performed using the Sequentially Numbered Opaque Sealed Envelope (SNOSE) method. At the time of treatment, the sealed envelope containing the subject's assigned individual treatment was opened. The same operator performed each intervention, and outcome assessment was conducted by a blinded assessor to prevent inter-individual variation and reduce assessment bias.

Intervention

Surgical Procedure

The area was anesthetized using 2% lignocaine hydrochloride with 1:80,000 adrenaline. A BONART electrosurgery unit was used for the procedure. The unit was set to the cutting mode (CUT), and the intensity was set at 6 RF/2 MHz. Using a fine wire electrode (T4), the frenum was excised up to the desired depth. The electrode tip was used in a shaving motion intermittently and kept moving while being irrigated with normal saline to allow adequate tissue cooling. This prevented heat buildup and unwanted tissue destruction. The test group underwent frenectomy by electrosurgery followed by the application of Gengigel® (HA gel), while the control group underwent frenectomy by electrosurgery without the application of Gengigel® (HA gel).

Parameters

The Landry et al. healing index [20], which provides a grading from very poor to excellent, was assessed at the first, second, third, and fourth weeks, and the visual analog scale (VAS) pain scale was assessed on postoperative days 1, 3, and 7. The VAS was recorded on a scale of 1-10 [11].

Assessment of wound healing

Wound healing was evaluated using the Landry et al. healing index [20]. This index assesses soft tissue healing based on five parameters: tissue color, response to palpation (bleeding), presence of granulation tissue, epithelialization of incision margins, and suppuration. Each clinical parameter contributes to a final healing score ranging from 1 to 5 (1: very poor healing; 2: poor healing; 3: good healing; 4: very good healing; 5: excellent healing). Higher scores indicate better healing of the surgical wound.

Postoperative care and instructions

The patients were advised to take amoxicillin 500 mg thrice daily, Hifenac P twice daily, and pantoprazole 20 mg twice daily for three days. They were instructed not to brush or disturb the site. They were informed that a whitish plaque may develop in the area where the frenulum was cut (due to secondary repair), which should not be disturbed and left in place, as this will serve as a "band-aid" to protect the surgical wound. This plaque will disappear in a few days. The subjects were recalled after two weeks for review. The periodontal dressings and sutures were removed after two weeks, and the site was irrigated with saline. All treated sites healed uneventfully. Oral hygiene instructions were reinforced.

Outcome measures

The study participants were followed up to one month postoperatively. Healing was assessed using the Landry et al. index, which provides grading from very poor to excellent at the first, second, third, and fourth weeks, while the pain assessment was performed using a VAS pain scale (1-10) on the first, third, and seventh postoperative days.

Data analysis

All collected data were entered in Microsoft Excel (Microsoft Corporation, Redmond, Washington, United States) and imported into Python (Python Software Foundation, Fredericksburg, Virginia, United States). Statistical analyses were performed using Python, and graphs were generated using MATLAB (The MathWorks, Inc., Natick, Massachusetts, United States). All quantitative variables are described with means and standard deviations. An independent t-test was used to compare changes between the two groups. For all tests, a p-value of <0.05 was considered statistically significant.

## Results

Twenty-four participants were recruited for the study. The mean age of the participants in the study group and those in the control group was 20.42 years and 20.5 years, respectively. There were seven males and five females in both groups.

The comparison of the healing index at different time periods showed that there was a statistically significant difference between both groups at baseline and days 7, 14, 21, and 28 (p<0.05). (Table 1).

**Table 1:** Comparison of the Landry et al. healing index between the test and control groups Independent t-test was used to compare the Landry et al. healing index between the test and control groups.

Time point	Test group (n=12) (mean±SD)	Control group (n=12) (mean±SD)	t-value	p-value
Day 7	2.50±0.52	1.67±0.65	3.46	0.002
Day 14	3.08±0.67	2.08±0.67	3.66	0.001
Day 21	3.92±0.67	2.50±0.52	5.78	<0.0001
Day 28	4.58±0.51	2.75±0.62	7.87	<0.0001

The comparison of perceived pain using the VAS at different time periods showed that the values were higher in the control group than in the study group (Table 2).

**Table 2:** Comparison of perceived pain between the test and control groups using the visual analog scale An independent t-test was used to compare the perceived pain between the test and control groups using the visual analog scale.

Time point	Test group (n=12) (mean±SD)	Control group (n=12) (mean±SD)	t-value	p-value
Day 1	1.92±0.51	4.25±1.60	-4.80	<0.0001
Day 3	1.17±0.39	3.33±0.65	-9.89	<0.0001
Day 7	0.83±0.39	2.42±0.67	-7.09	<0.0001

## Discussion

Wound healing is a dynamic process that occurs by primary or secondary intention. Primary intention healing occurs when wound margins are closely approximated, as in scalpel incisions, whereas secondary intention healing occurs when the wound edges are not approximated and the tissue repair process progresses from the wound. Periodontal wound healing is inherently complex because of the structural composition of the periodontium [21]. Furthermore, the oral cavity is exposed to a contaminated environment with a significant bacterial load, making predictable regeneration more challenging. Consequently, healing outcomes following surgical procedures may vary significantly. Electrocautery produces a surgical incision with minimal bleeding, similar to that produced by lasers. Electrocautery is safe and effective; therefore, it is recommended for frenectomy. It causes minimal postoperative bleeding with minimal complications. According to clinical research, patients who undergo frenectomy using electrocautery report better postoperative discomfort and function than those who undergo a scalpel approach. Given its cost-effectiveness and procedural efficiency, electrocautery remains a practical alternative to laser-assisted frenectomy [22,23].

Pain perception after a surgical procedure is subjective and is influenced by individual thresholds. Among the available assessment tools, the VAS is widely accepted because of its simplicity, reliability, and reproducibility. Postoperative discomfort is common after periodontal procedures and may affect patient satisfaction and quality of life, making its evaluation clinically relevant [24].

In the present study, the demographic characteristics of the study and control groups were comparable. Assessment of the healing index at baseline and on days 7, 14, 21, and 28 revealed significantly improved healing in the HA group compared to that in the control group. The enhanced healing observed in the HA group may be attributed to its biological functions during tissue repair. HA is a naturally occurring glycosaminoglycan abundantly present in nonmineralized periodontal tissues and plays a crucial role in extracellular matrix stability, cellular migration, angiogenesis, and modulation of inflammatory responses.

During the granulation phase of healing, the degradation of high-molecular-weight HA produces low-molecular-weight fragments that stimulate angiogenesis and tissue remodeling. Previous clinical studies have supported the beneficial effects of HA in periodontal and oral surgical procedures [25-29]. Topical application of 0.2% HA has demonstrated improvements in gingival parameters, including a reduction in plaque accumulation and gingival bleeding. However, variability in outcomes across studies suggests that concentration, frequency of application, and treatment protocol may influence clinical effectiveness.

In the present study, postoperative pain was assessed at baseline and on the first, third, and seventh days. Pain scores decreased progressively in both groups over time, consistent with the natural healing course. However, the HA group reported significantly lower pain levels than the control group. The anti-inflammatory effects of HA, along with its ability to maintain tissue hydration and stabilize the wound environment, may explain the reduction in postoperative discomfort observed in this study.

In summary, these biological properties explain the superior healing outcomes and enhanced patient comfort observed with adjunctive HA application following electrocautery-assisted frenectomy. The scope of this study demonstrated the beneficial effects of adjunctive HA use on soft tissue healing and postoperative pain reduction. These findings suggest that HA may be a valuable adjunct in periodontal surgical procedures aimed at optimizing clinical outcomes and patient comfort.

Limitations

Despite these promising findings, certain limitations must be acknowledged. The sample size was relatively small, which may limit the generalizability of the results. The follow-up period was restricted to 28 days, and the long-term stability of the clinical outcomes was not evaluated. Histological analysis of wound healing was not performed. Additionally, pain assessment using VAS remains subjective and is influenced by individual variability. Therefore, future studies with larger sample sizes, longer follow-up periods, and histological evaluations are recommended to further validate these findings and establish standardized protocols for HA application in periodontal surgery.

## Conclusions

The adjunctive application of topical HA following electrocautery-assisted frenectomy resulted in significantly improved soft tissue healing and reduced postoperative pain compared to electrocautery alone. Statistically significant differences were observed in both the healing index and VAS scores at multiple postoperative intervals, favoring the test groups. The results of the study indicate that HA speeds up the healing process and reduces the perception of pain in treating frenectomy with electrocautery.
